# Could wound care benefit from the artificial intelligence storm taking place worldwide

**DOI:** 10.1111/iwj.14171

**Published:** 2023-04-11

**Authors:** Douglas Queen

**Affiliations:** ^1^ Editor

The buzz around artificial intelligence (AI) has been covered in previous editorials.^1^, ^2^ In the editorials, I discussed how it was helping create and drive data driven wound care practices. This was expanded upon in a recent editorial by Cross and Harding.^3^

Since these editorials were written, the buzz has certainly become louder, even if only in the public rather than the professional domain. AI has the awesome power to change the way we live our lives, in both good and dangerous ways. But are those in power prepared for what's coming, similar to where we find ourselves today with social media and any impending regulation.^4^ Let us hope so!

Chatbots and AI‐assisted search are the new buzz within the AI field. You may have heard of Chatbot‐GPT and how it will revolutionise our search capabilities, even with the potential to write up our research findings. Perhaps even writing your next IWJ paper. Students and researchers, particularly where English is not their first language, are excited. Teachers, assessors and reviewers are less excited. From writing stand‐up comedy scripts to poetry and even generating visual images regarded as art, AI has come a long way recently. Machines, at least to the general public, have achieved creativity.^5^

Regulators and governments recognise this is the emergence of an extremely powerful technology; however, it can be used for both good and bad, and as such, it will require close monitoring and possible regulatory review. One of the main dilemmas is that many of the players in AI are the major tech companies, which are currently under scrutiny regarding social media regulation. The potential downsides of AI include the automatic creation of an ocean of disinformation across multiple social media outlets. The biggest concern is not its generation, but rather that they would be entirely believable and perceived to come from legitimate accounts. Even if the government succeeds in enacting new social media regulations, this may be pointless in the face of a flood of pernicious AI‐generated content.

To date, millions of individuals have used CHATGPT over a relatively short time frame. Others are waitlisted to do so. Large corporations are paying attention and investing as necessary; for example, Microsoft's Bing search engine already uses this approach, and the company has invested $10 billion to do so. ChatGPT answers questions using natural human‐like language, and it can also mimic other writing styles, such as those of songwriters and authors, using the internet as it was in 2021 as its knowledge database. So, this rapid adoption and growth is both serious and a seismic change in the adoption of AI.

This success has resulted in OpenAI quickly releasing GPT‐4, the latest version of its hugely popular artificial intelligence chatbot. These chatbots are a type of generative artificial intelligence that uses algorithms and predictive text to create new content based on prompts.

The ultimate aim of chatbots lies in internet search, where many pages of web links can be replaced with one definitive ‘intelligent’ answer. We all know the ultimate go to search engine is Google. Google is launching an AI‐powered chatbot called Bard to rival ChatGPT.^6^ Bard is built on Google's existing large language model, Lamda, which one engineer described as being so human‐like in its responses that he believed it possessed consciousness.^7^ The tech giant also announced new AI tools for its current search engine. Generative AI is our future. As search engines like Google became the norm and social media entered every aspect of our lives, generative AI is the next evolutionary phase of the integration of mankind with technology.

Some companies are working with generative AI to provide research communities of practice where researchers can both interact with and share research content.^8^, ^9^, ^10^ Through such communities, where personalised content becomes the individuals personalised data/study repository, this provides a personalised pool of information from which generative artificial intelligence can provide a more personalised output to your search or question. These more specialised repositories provide a more specialised pool of research than the current ‘total internet’ approach of products like ChatGPT. This will provide efficiencies for researchers, saving significant time spent currently sifting through a mountain of research to find those of interest.

So, what could this mean for wound care? Wound care, like any health care arena, is data rich. Wound care, is perhaps one of the most data rich areas because of its interaction with or relationship to multiple human comorbidities. AI is certainly going to revolutionise this data management and aid with both assessment and treatment approaches.^3^ Generative AI, however, can help understand this complexity and provide “accurate” summaries of data and outcomes. It can help provide summaries of individual and multiple studies, helping researchers not only understand the content but also provide the outputs to educate others.

Imagine this scenario. You want to write a historical review of wound bed preparation, both its development and application. An initial Google Scholar search displays nearly 10 000 articles for those search terms. Fortunately, a quick Pubmed search shows nearly 400. So, let us run with the latter, as it seems more manageable; however, it is still a mighty time‐consuming task to review and summarise 100s of papers to begin your review article. Imagine if you had a reliable, trusted community of practice that uses an AI approach that would summarise the papers for you, either individually or collectively. Taking it a step further, a generative AI component could then use these summaries to start to draft elements of the review for you to craft into an article. This is simply the automation of laborious research processes. Often, the more senior researchers use the more junior researchers to complete these preliminary aspects. Imagine a world where a machine does so in mere seconds! This would revolutionise research by providing efficiencies that would free up time to do actual research rather than spend significant time and money doing the more administrative parts.

All I can say is watch this space! This approach is not too far in the future, and some would even argue the capability exists today; it just requires a change in attitude or thinking to implement.^11^ Think social media resistance several decades ago and how reliant we are on it today.^12^
